# The Organic Food Choice Pattern: Are Organic Consumers Becoming More Alike?

**DOI:** 10.3390/foods10050983

**Published:** 2021-04-30

**Authors:** Fernando Nunes, Teresa Madureira, José Veiga

**Affiliations:** 1 Centre for Research and Development in Agrifood Systems and Sustainability, Instituto Politécnico de Viana do Castelo (IPVC), 4900-347 Viana do Castelo, Portugal; fnunes@esa.ipvc.pt; 2 Escola Superior Agrária (ESA), Instituto Politécnico de Viana do Castelo (IPVC), 4900-347 Viana do Castelo, Portugal; 3 Escola Superior de Tecnologia e Gestão (ESTG), Instituto Politécnico de Viana do Castelo (IPVC), 4900-347 Viana do Castelo, Portugal; jmcveiga@estg.ipvc.pt

**Keywords:** organic food, attributes, bestworst scaling, consumer behavior

## Abstract

There is no doubt that the search for organic products is already more than a trend; it is an indisputable reality. More and more people are opting for a healthier lifestyle that starts with food, which has awakened a growing interest in understanding the reasons for these purchases. The motivational attributes of consumers’ decisions regarding the consumption of organic products are the main aim of this study. The survey included 250 respondents that filled a questionnaire by email and by personal interviews. We used a non-probabilistic sampling method, namely convenience sampling and the best–worst scaling method to analyze 10 attributes of organic purchasing decisions. Then, we studied the impact of the classification variables age, gender, academic level, place of residence, children under 18 living at home, and place of purchase of organic products on the attributes. Applying a chi-square test, we only obtained statistically significant differences for children under 18 living at home and the certification warranty (*p* = 0.011). The results show the dominance of credence attributes and egoistic motivations on organic consumption and may indicate a path towards the standardization of the organic consumer profile. This study emphasized that we may be facing a new organic consumer, for whom health-related factors are not just significant but overwhelming as well.

## 1. Introduction

Demand and consumption of organic products has increased significantly in recent years [1,2,3,4], triggering significant and sustained growth on the supply side of these products. Portugal has followed this worldwide trend in consumption, also accompanied by a significant evolution in production, with the number of organic producers in Portugal almost doubling, from 2434 to 4267 between 2010 and 2017 [5]. Growing consumer interest and, consequently, the development of an increasingly attractive market segment has motivated several researchers to learn, characterize, and explain the behavior and motivations of organic consumers [1,2,3,4,6,7,8,9,10], which is fundamental for the sustainable growth of companies, in an increasingly competitive market segment [4,11]. One of the most cited reasons for the growing increase in demand for organic products is differentiation based on intangible attributes [12,13]. Indeed, some consumers essentially base their choices on their perceptions that these foods are healthier, more environmentally friendly, and have better nutritional qualities [3,4,14]. These attributes, namely credence attributes, although they cannot be objectively evaluated even after purchase or consumption, play a very relevant role in the purchase decision process. There is some consensus regarding the importance of the healthy attribute (and consequently, the perception that these products are safer), making it the most relevant predictor of attitudes and behaviors associated with the consumption of organic products [1,15,16,17,18,19,20,21]. Environmental awareness is an increasingly present theme in the political and social debates of developed countries, and it has the power to lead more and more consumers to choose products and brands based on aspects relating to environmental protection or animal welfare [14,17,22,23,24,25]. Moreover, another essential credence attribute relates to the belief that organic food, in addition to being healthy, contains healthy nutrients [26,27,28].

Beyond the credence attributes (healthier, more environmentally friendly, and with more nutritional qualities), there are other attributes that are equally important for the evaluation of organic products, which, unlike credence attributes, can be objectively observed and measured, before or after purchase or consumption. These are defined as demand and experience attributes, and they can be assessed and judged, respectively, before and after purchase or consumption [1,29,30].

Food taste is the most crucial attribute of experience, as consumers associate organic products with tastier foods than conventional ones, since they are produced in a more natural way [4], and therefore have a cleaner taste [17,31].

With regard to the search attributes that are observable and objective prior to purchase, the lower availability of organic products in traditional retail circuits, together with a normally higher price, constitute a challenge for the development of positive attitudes towards organic products [4]. The usually higher prices of organic products are a barrier to consumption, but it may decline with improvements in distribution channels for organic food [32]. A new profile of consumers has been identified, more pragmatic and less motivated by ideological issues, for whom price is a determining factor in the purchase of organic products [17]. These consumers purchase organic products only when the price difference between these products and conventional products is minimal [33]. The availability of organic products is a relevant attribute in most markets, since the difficulty of accessing these products can make them difficult to find or even prevent their purchase [34]. Indeed, consumers usually do not like to spend a lot of time looking for green products [35], preferring products that are easily available [36]. For many consumers who are more pragmatic in the purchase decision process, the unavailability of organic products in their purchase routines can lead to the purchase of conventional products. However, as a driving factor for purchase, certification guarantees can help insofar as they increase consumer confidence [4,9,17,32,37]. Most food products have no indication as to the presence of genetically modified organisms (GMOs), and it is not possible for consumers to gauge their presence in the food they consume. In organic farming, the presence of GMOs is regulated, and their use is restricted to specific legal limits. The certification of organic products, thus, acts as a guarantee of the absence of GMOs [1,38]. The origin of organic products is another important factor, especially for consumers with ideological and ethical motivations. This aspect is associated with a guarantee of fresher seasonal foods, with superior quality and with lower environmental impacts due to transportation [17,39,40]. In fact, organically produced food has become part of the globalization process as demand has increased further, and it cannot be met by national supply alone. Local food, by definition, represents an opposite trend, leading to more proximity in food production [41].

If we analyze retrospectively previous studies regarding consumer behavior toward organic products, we can detect some changes in the perception and appreciation of the main attributes associated with purchase and consumption. From a supply perspective, over the last few years, the availability of these products has led to numerous points of sale, and today, it is common to have organic supermarkets in various locations. This evolution, both in demand and in supply, may indicate the emergence of a “new organic consumer.”

In this study, we used the best–worst scale (BWS) method to collect and analyze the data. Among other benefits, the answer consistency at the individual level helps to explain the superiority of the BWS method for classification, clustering, and prediction in relation to the rating scale methods [42]. As far as we are aware, no study on the buying behavior of organic products, in Portugal or abroad, has used the BWS method to date. Therefore, its application allows a new and accurate approach and can provide relevant information as well.

The main purposes of this investigation are to understand the perceptions of organic consumers and to analyze the motivations and barriers to their consumption in order to update and characterize their consumption profile.

This paper is structured in six sections. After the introduction, we address the methodology, including a description of the questionnaire design, the sampling method and survey administration. The results are detailed in Section 3, followed by the discussion in Section 4. Our conclusions are presented in Section 5 and, in Section 6, we outline the limitations of the study.

## 2. Methodology

### 2.1. The Best–Worst Scaling Method and Its Application

Rana and Paul (2017) identified some striking studies based on the performance of empirical tests that involved the participation of consumers [43]; surveys and questionnaires that were analyzed through correlation and regression methods [44]; ordered probit models [45,46]; descriptive statistics, chi-square, ANOVA, and factor analysis [31,47]; and finally, descriptive statistics and non-parametric tests such as Mann–Whitney and Kruskal–Wallis tests [48]. More recently, relevant studies on buying behavior for organic products have used fuzzy theory [49], the theory of planned behavior [50], focus groups [39], and, above all, Likert-type scales [1,24,26,51,52]. Other authors have drawn attention to the limitations of methods based on the simple ordering of attributes, such as Likert-type scales, in terms of the difficulty of interpreting and validating new attributes and the impossibility of conducting comparisons among them [53]. To avoid the bias inherent in simple ordering methods, several authors have applied processes based on discrete choice (scaling methods) that allows consumers to set the level of preference for a particular attribute [54,55,56].

The BWS method has become a popular way of studying how important a particular issue is to an individual or group of individuals relative to other issues under consideration [57]. The BWS method was introduced by Finn and Louviere (1992), who used it to measure public concern about food safety. The BWS method has since been used in numerous and diverse searching backgrounds, including animal welfare [58]; landscape architecture [59,60]; elderly well-being [61]; perception of success in professional carriers [57,62]; corporate social responsibility [63]; consumer behavior towards agri-food products [64,65,66]; consumers’ functional application (app) requirements [67]; and above all, health care [68,69,70].

The BWS method has gained in popularity based on the idea that this approach has greater discriminatory power than other scale measures [71] and allows for better comparisons among countries and segments [72]. Rather than asking respondents to rate items one at a time, respondents are shown a predefined number of candidate items and are asked to choose the two items within each set that they consider to be the best and worst [53].

Two main advantages have been identified for adopting a BWS methodology. First, the method involves a fairly simple task for respondents and it is less cognitively demanding to select extremes on a scale than to rank all items simultaneously [57,73,74]; second, it produces rich information to the researcher by providing sufficient information to calculate even individual-level scales and to provide precise and comparable scales [57,69,74,75].

### 2.2. Questionnaire Design

A two-part questionnaire was developed for this study. The questionnaire was prefaced by an explanation that its purpose was to understand the buyer behavior of organic consumers. The first part of the survey included the following six classification variables: age, gender, education level, place of residence, children living at home, and place of organic product purchase. The second and main part of the survey was designed to measure the importance that respondents attached to specific attributes of organic products using the BWS method. Ten specific attributes of organic purchasing decisions were selected (Table 1). These main attributes were chosen based on the previous literature review, and included the following: three credence attributes, i.e., health benefits [15,16,21], environmental impact [24,25], and nutritional value [27,28]; one experience attribute, i.e., expectation of better taste [31]; and six search attributes, i.e., price [17,32,76], more natural appearance [46,77], certification warranty (EU logo) [9,32,78], origin [39,40], availability [50], and absence of GMOs [1].

The ten attributes were combined into ten choice sets of four items each, and respondents were asked to select the best and worst attribute in each set, i.e., the most and least important attribute of the decision to purchase organic products. Four or five items per set are regarded as optimal for respondent evaluation since a greater number could lead to respondent fatigue [79]. The question sets were balanced in factor frequency, positional frequency, and orthogonality. Therefore, each attribute appears the same number of times across all choice sets, and each pair of attributes appears only once within each set. Multiple versions of the survey were generated to increase variation in the position and combination of attributes across respondents, thereby reducing any potential context bias [79].

### 2.3. Sampling Method and Survey Administration

In this study, we used a non-probabilistic sampling method, namely convenience sampling. This method is widely used and consists of selecting a sample whose elements are familiar to the subject under study. The sample elements were selected because they were the most appropriate and not because they were selected by a statistical criterion. In this case, most respondents were recruited based on their access to the web (approximately 70% of the sample elements), and some were interviewed in person (the remaining 30%). Therefore, most questionnaires were sent by email. This type of technique usually results in a high number of non-responses and even with incentives, the response rate often does not reach 10% [80]. To overcome this problem, personal interviews were carried out to maintain a balance in the sample based on demographic characteristics. Generally, this type of sampling is more accessible to apply than other methods, and it has lower costs. We used Sawtooth Software (Sawtooth Software, Provo, UT, USA.) to design the survey, implement questionnaires and to analyze the data. The survey links were first distributed through email (the questionnaires were written in the Portuguese language), and complimentary in-person interviews were undertaken in open-air organic markets. We also requested participants to contact family members, friends, and colleagues living in the three selected Portuguese regions to participate in the survey. These regions were chosen because they are in the metropolitan areas with the highest population densities of the country. Regular consumers of organic products, that is, those who consumed at least three categories of organic products per week (vegetables, fruits, dairy products, meat, groceries, etc.) were only considered in the survey. Nevertheless, as this is an exploratory study and the BWS method is considered to be highly robust, data can be discussed and analyzed, offering several preliminary findings for works to come. This type of study certainly contributes to the cumulative development of knowledge and is a necessary and sufficient method that holds up well as compared with other methods [81]. From a total of 520 responses, only 250 were complete, and the remaining participants (270), most people residing in *Big* Lisbon, were excluded on the grounds of incompleteness. The survey took place from January to April 2019, and the summary demographic data are presented in Table 2.

## 3. Results

### 3.1. The Best and the Worst Preferred Attributes

We began by computing best–worst raw scores for each respondent for each organic food characteristic. The number of times each item was chosen as most important (best) and least important (worst) were summed up across all choices, and the worst were subtracted from the best, resulting in best–worst raw scores. Because best–worst raw scores are often perceived as difficult to understand, they are often rescaled to allow for an easier and more intuitive interpretation [82]. Thus, the best–worst raw scores were rescaled, or transformed, into rescaled scores (0 to 100 scaling) so that the scale presented ratio-scaled probability properties with the sum of all items being 100. This assumes that an item is chosen a particular percentage of times as compared with other items [79]. Table 3 presents the analysis of the attributes with 95% confidence intervals for the rescaled scores averages, ranging from the most important/strong attribute to the least important/weak attribute.

**Figure 1:**
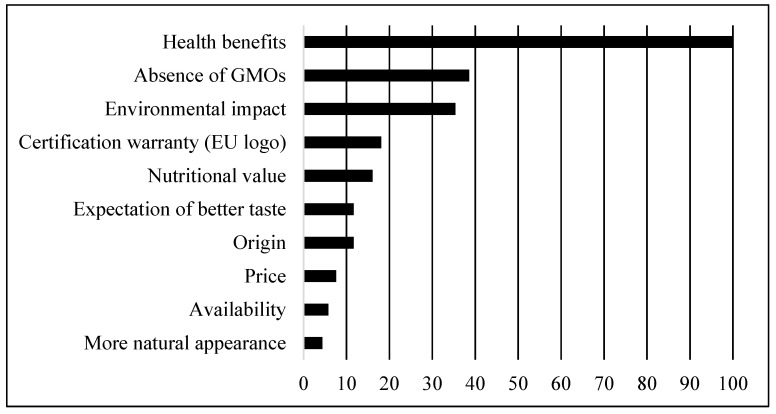
Standardized ratio scale relating to the 10 organic attributes.

For convenience and better perception, a graphical analysis of the attributes was performed using the standardized ratio scale. Data were sorted by decreasing level (Figure 1).

The purchase of organic products seems to be conditioned mainly by health benefits resulting from the consumption of these products (100.0). Indeed, the second most chosen attribute, absence of *GMOs*, has an influence on organic consumption (38.6) that is far less strong than that of the first attribute, health concerns. The environmental impact resulting essentially from organic production completes the group of the three main attributes that influence the choice of organic products, and it is rated third in the buying process (35.4). The certification warranty, nutritional value, origin, and expectation of better taste attributes form a middle group of four elements with intermediate influence on organic consumption, registering standardized ratio scale values ranging from 11.7 to 18.1. To conclude, we can consider the existence of a third and final group of three attributes with reduced capacity to influence organic consumption, i.e., price, availability, and more natural appearance.

### 3.2. Impact of Classification Variables on Attributes

To analyze the importance of the relationship among the attributes and the classification variables age, gender, academic level, area of residence, children under 18 living at home, and place to purchase organic products, first, the continuous scale of each attribute was converted from the lowest rescaled score to the maximum rescaled score on a four-point Likert-type scale. For this, three cut points (thresholds) were considered, and the rescaled scores of each attribute were grouped in four ordinal and mutually exclusive classes. Then, a chi-square test was applied for independence between the attributes and the classification variables. However, some assumptions for the application of this test failed, i.e., some cells had expected counts less than one and, in many cases, more than 20% of the cells had expected counts less than five. To correct this problem, we combined the classes of some classification variables to increase expected counts, as well as used Monte Carlo simulation techniques (Table 4).

Table 5 presents the significance of the relationship among the attributes and the classification variables. The cells marked with “a” mean significant relationships (*p* < 0.05) and cells marked with “b” mean marginally significant relationships (0.05 < *p* < 0.1).

Table 5 shows that there are no statistically significant differences between any of the ten attributes and the classification variables age, gender, academic level and area of residence. However, there are statistically significant differences (*p* = 0.011) between the classification variable children under 18 at home and the attribute certification warranty (EU logo), the latter being a specific characteristic of the organic products. In what concerns the classification variable children under 18 at home, there are also marginally significant relationships with the availability attribute, being *p* = 0.052. Likewise, we can also identify a marginally significant relationship between the place to purchase organic food products and a more natural appearance (*p* = 0.053).

## 4. Discussion

### 4.1. Driving Influencers of Organic Buying Behavior 

The main objective of this study was to analyze a set of attributes that influence the purchasing behavior of consumers of organic products in Portugal. The results of the best–worst analysis have been presented, along with a correlation between classification variables and ten specific organic attributes.

By applying a best–worst standardized ratio scale, we determined that health benefit is the most important attribute in the organic food buying process. There is a huge debate about the relevance of health as a predictive factor in food consumption in general [83,84], and for organic product intake in particular. De Canio and Martinelli (2009), through a structured questionnaire applied to organic consumers in northern Italy, concluded that it was not possible to find any effect relating to health issues. In a recent study with the opposite conclusions, Tandon et al. (2021) analyzed the behavior of consumers of organic products in Japan and concluded that health concerns significantly affected the acquisition of these types of products. However, the influence of health concerns was two-fold and antagonistic, given that it influenced both facilitators (despite being the majority) and inhibitors of organic consumption. Although the concept of health can have different interpretive contexts for consumers of organic products [15], and the consumption of organic foods is often considered to be the result of a complicated set of interactions between the organic label, type of food, and health control of the individual [32], our study indicates a clear prevalence of health concerns as the main attribute with regard to the consumption of organic foods. These results were also confirmed in a seminal literature review that identified, for a period of 25 years, a set of 150 studies in which the health benefits were indicated by the majority of consumers (66%) as the leading cue in the purchasing process of organic products [1]. The relevance of the health benefits attribute is also in line with other studies that have shown that consumers’ organic identity and purchase behavior were positively driven by health concerns and food safety [4,15,16,21,25,85,86,87,88]. Using a multilevel meta-analysis applied to studies on consumption of organic products (mainly grocery, milk, fruits, and vegetables) over the past 25 years, Rana and Paul (2020) recently concluded that health issues outweighed all other attributes with regard to factors that mainly affected bio consumption [89]. These results strengthen the overall perception that credence and egoist attributes as health benefits are assuming an increasingly important role in food consumption [50].

The absence of GMOs was classified in second place, but its predictive force in the choice of organic products was just over a third as compared with health issues. Considered as correlated with and relevant to consumers’ preferences for buying organic products [85], the health benefits and the absence of GMOs items counted, in this study, for more than half of consumers’ preferences (standardized importance weight of 55.6%). Opposition to the consumption of genetically modified foods in Europe is not a recent trend. A study carried out in six European countries (France, Germany, the UK, Italy, Poland, and Portugal) revealed that a third of the population in these countries would not use any type of food with GMOs [90]. More recently, several studies [91,92,93,94] have continued to emphasize the importance of the presence or absence of GMOs as a factor in food choice and consumption. This assertion being true, which the present study brings as a novelty, is the fact that the strength of this attribute, although significant, is far less robust than health concerns, chosen as the most critical factor.

A recent study conducted in Vietnam concluded that, for emerging markets, environmental concerns definitively drive consumption and attitudes towards organic food [24]. In India, another emerging country, environmental consciousness does not moderate the associations between different users’ motivations and organic food buying behavior [95]. In Switzerland, the environmental impact of organic meat production is considered to be a more significant attribute than health concerns [40]. The results of our study do not follow the previous trends, since the environmental impact of organic production ranked third in the set of the most relevant factors in the organic purchase process. Thus, our findings are closer to the results of other authors, who clearly prefer the domain of selfish motives over altruistic motives, suggesting that environmental consciousness (an altruistic motive) is not related to organic food identity, at least for mature organic markets such as the Danish one [16]. Somehow, in this respect, the diversity of conclusions is high, and this may be based, in part, on the degree of development of the analyzed markets in each study, namely, on the way in which the importance of environmental issues is valued or perceived. In our case, it is necessary to be aware that the BWS method does not reflect a logic of absolute values but instead, a relative weighting of the items considered. This means that although the environmental impact attribute is essential, respondents considered its relative persuasive power to be much lower (35.4) than health concerns (100).

Certification warranty (EU logo), nutritional value, origin, and expectation of better taste can be grouped as a set of attributes with medium to low capacity to define the main choices of organic consumers. The reduced impact of the European Union organic logo as a guiding element for bio consumption, identified in this study, is in line with the work of other authors, who found that consumers are willing to pay a premium price for other EU quality products than organic food [96]. Equally, the European organic production logo is also associated with situations of misinterpretation and erroneous claims [97], and therefore its role is increasingly subject to criticism and less appreciation.

Surprisingly and unexpectedly, the expectation of better taste attribute has a reduced ability to influence the consumption of organic products. Although in terms of taste perception, there may be considerable consumer heterogeneity [98], a significant body of literature refers to (better) taste as inextricably associated with organic products [4,39,45]. However, it is important to consider the recent growing relevance, in organic consumption, of credence attributes as compared with experience attributes, and therefore these results should not be considered immediately contrary to the mainstream. Finally, it is also possible that organic products are evaluated as higher in perceived taste due to their increased healthiness [99], which would allow us to infer that consumers could, in the presence of both attributes, choose the health benefit predictor as being more relevant.

Although some studies have considered the origin (of production) as a relevant driving factor in organic-minded consumers [41], our findings are in line with those of Ditlevsen et al. (2019), who, on the one hand, pointed out the reduced relative importance of the origin of production as a predictive element in the choice of organic products [15]. In other words, the theme is still relevant, but other attributes are more impactful. On the other hand, one may argue that a huge number of European consumers consider that the negative environmental impact resulting from the import of many organic products is obviously a consequence of the great distances these products travel from their countries of origin (mainly Asian) to the European markets. Thus, it is possible that some respondents have included their concerns about the origin of the organic products when choosing the environmental impact attribute. Given that, in the BWS method, the hierarchy of attributes reflects their relative importance, the results mean that although origin is an important attribute, environmental issues are much more relevant. In fact, the latter have approximately three times more predictive power for the consumer than the origin (35.4 vs. 11.7).

In a set of 100 responses, the possibility of respondents choosing the origin of the products or the expectation of a better taste as the most important attribute was only 11.7 for both. This can be interpreted as an unexpected diminishing of the importance of these attributes in the choice of organic products.

The so-called search attributes, i.e., price, availability, and appearance have less influence over the choice of organic products, with standardized ratio scores of 7.6, 5.8, and 4.4, respectively (100 being the maximum score). Unsurprisingly, in Western countries, price is not considered to be a top factor in the set of attributes that guide the consumption of organic products. Indeed, the willingness to pay a premium price for organic is often high. Therefore, its relative importance in the rationale of consumers decreases [32,39,100]. In some cases, the consumption of organic products is even positively conditioned by the practice of prices that are significantly higher than those for conventional food [1]. In line with these assertions, some authors found that retailers should be very judicious in implementing discount policies on organic products, as this could lead to negative effects on sales [101,102].

The dominant results in the literature point out that availability is, in effect, an important conditioning factor in the purchase process of organic products. Thus, and despite our findings not following this trend of analysis, we believe that we must be cautious in self-criticism. Indeed, it should be emphasized again that respondents are forced to choose between attributes (instead of being asked to classify them), resulting in the low relative position of the availability attribute as compared with other more impactful attributes, such as the case of health concerns and the environmental impact of organic production.

A more natural appearance attribute occupied the last position as a significant predictor of organic food buying intentions. This result contradicts the apparently erroneous perception that the consumer is often driven by extrinsic attributes that manifest strong visual appeal and attractiveness. When the dichotomy between the look of conventional and organic products was more accentuated (and explored), the appearance of organic products was considered to be an important predictor of purchase intentions. However, nowadays, its relative weight is lower and tends to diminish [103,104,105]. Currently, supported by our findings, we believe that, among other predictors, the relative strength of the visual appeal of organic products is, in fact, much less powerful than it was a few decades ago.

### 4.2. How Models of Personal Categorization Influence Organic Consumption 

The impact of the classification variables on attributes shows very few indicators with significant correlations. This might mean that these ten attributes are not affected by classification variables such as age, gender, academic level, or area of residence. However, we identified a strong relationship between the existence or absence of children under 18 at home and the attribute guarantee of certification (EU logo).

Although one study [106] called into question the impact of the presence of children at home and the effect on the purchase of organic products, a significant number of studies also mentioned the high probability of relatedness between having children at home and the pursuit of certified quality food, as is the case with formal organic products [107,108,109,110]. Although this relationship may change according to the number of children in the household and their age, the findings are consistent in pointing out parents’ concerns to give their children safe food, thus, protecting their health. The emphasis on the guarantee of the intrinsic quality of the food consumed justifies the concern of parents and guardians about the demand for certified food. In this context, and when it comes to the nutrition of the youngest, the demand for food with a quality guarantee attested by independent and credible entities, as is the case with organic products, is fully justified, and our results are in line with other relevant studies [17,49,103,111,112].

Studies also identified a marginally significant relationship between the place to purchase organic food products and the more natural appearance attribute. The analysis of 89 empirical studies published between 2005 and 2018 on the obstacles and motivations that frame the consumption of organic products showed that 53% of these studies reported availability as a major barrier to consumption [45]. There is a new sort of consumer, mostly women with children, for whom shopping has to be an easy process [17]. The presence of children in a family influences and is influenced by the parents’ eating habits [113]. Likewise, several studies have pointed out that the greater or lesser ease of access to food products significantly affected their intake by children [114,115]. In this context, it is acceptable to confirm the findings of our study in the sense of the existence of relations that tend to be relevant between the presence of children at home and easy access to organic foods. Although not very strong, this correlation highlights the fact that the proximity between family houses and places where organic products are sold can contribute to a greater consumption of this type of product by families, particularly when they have dependent children.

A significant marginal relationship between the place to purchase organic food and the more natural appearance attribute was also identified. Although, as mentioned above, health concerns are often the determining factors in organic consumption, hedonic factors such as smell, attractiveness, and appearance, are increasingly highlighted as significant predictors of organic food buying intentions [116]. Effectively, consumers of organic products are conditioned by their appearance [45], and retailers should implement contexts and appearances in their stores that are as close as possible to the natural forms of production and presentation [101]. The correlation between purchasing venues and the sensory appeal of organic products, therefore, seems plausible to us. That is, more conservative and traditional sales outlets may be associated with organic products that appear to be closer to the natural way; conversely, more modern sales outlets, such as centralized retail, could offer consumers organic products with a more attractive and artificial appearance.

## 5. Conclusions

Our results may suggest the beginning of a path towards a standardized profile of frequent consumers of organic products. This assertion is based on two fundamental findings. The first concerns the number of consumers who consider the attribute health benefits as the most relevant when choosing organic products. In fact, the comparison between the number of times (609) this attribute was selected as best/more important, as compared with the second attribute, absence of GMOs (356), reveals its enormous importance as a predictive factor in the process of buying organic products. Furthermore, if we consider the standardized ratio scale index, which translates the relative strength of each attribute, we find that the second chosen predictor has the ability to influence the organic consumer that is only slightly more than a third of the strength of the strongest attribute (38.6 over 100). Additionally, the analysis of the standardized importance weight indicator reveals that the first two attributes (health concerns and absence of GMOs), account for more than half of consumers’ choices (55.6%). These data point to a polarization in the referential framework of the organic consumer profile, mainly conditioned by the relevance of issues related to health (a credence and egoistic attribute).

The second finding concerns the results of the correlations among the classification variables and the ten attributes. Except for the significant correlation between certification warranty (EU logo) and having or not children under 18 at home (*p* = 0.011), no other meaningful relationships were identified. This means that the different categories defined within each classification variable do not differ significantly as to how they value each attribute. This may mean, for example, that men and women are equally concerned about health issues, that people over 55 do not distinguish themselves from the youngest in terms of price sensitivity, or that respondents with higher education levels are just as sensitive to non-consumption of GMOs as those with a lower degree of education. Furthermore, it is essential to note that the three attributes where it is possible to identify significant relationships with the classification variables (certification warranty, availability, and appearance) were classified by respondents as having reduced or much reduced predictive strength in organic consumption. The nihilistic possibility of relatedness between attributes and classification variables might indicate a path towards a more homogeneous and global organic consumer, at least in consolidated markets.

The standardization of the organic consumer profile can be based on the consolidation of some characteristics of organic intake over others, that is, the prevalence of selfish and personal reasons for consuming organic food, similar to health concerns. We still do not have an unambiguous body of knowledge that confirms we are facing a new organic consumer. However, the increasing importance of the health concern attribute in organic food consumption predictors should be highlighted.

The results of this study may be helpful from a business point of view, especially in terms of production and distribution. On the one hand, producers should focus on agricultural production issues that guarantee, unequivocally, the obtaining of safe and healthy food. In this context, particular attention should be drawn to preventing contamination with GMOs. On the other hand, retailers must especially highlight and publicize the confirmed benefits of organic products from the point of view of their contribution to the promotion of consumers’ health. Again, it is essential to disclose the absence of GMOs in organic products.

## 6. Limitations and Future Avenues for Research

The main limitation of the present study lies in the impossibility of statistical inference, since the sample does not guarantee the extrapolation of the results to the whole of the national territory. To a large extent, the robustness of the BWS method compensates for this weakness. The different ways of collecting information (online and in-person) may have resulted in some bias, since the contact conditions were different (in the comfort of the house or in an open-air market). In this context, the time available to respond to the survey (a determining variable in the quality of the data) may have been slightly different among respondents.

It can be inferred by the present study that we may be facing significant change in the ordinary organic consumer profile. Considering that this is a new statement, it needs further investigation. Thus, we consider that new comparative works are necessary to attest to the validity of our proposals, which must be undertaken at a national level, but above all, within the framework of comparisons with other countries. In this sense, the same survey and the same research methodology must be implemented abroad, promoting comparisons among nations that the BWS method allows. In any case, efforts must be made to ensure that the sample is as widely representative as possible so that the conclusions are more accurate. Finally, we also believe that it would be very useful to carry out a horizontal comparative study which would ask regular and non-regular consumers about the same attributes.

## Figures and Tables

**Table 1:** Organic attributes.

**1**	Price
**2**	More natural appearance
**3**	Certification warranty (EU logo)
**4**	Origin
**5**	Expectation of better taste
**6**	Availability
**7**	Health benefits
**8**	Environmental impact
**9**	Nutritional value
**10**	Absence of GMOs

**Table 2:** Summary demographics for survey interviewees.

Classification Variable	Modality	*N*	
V.1—Age	15–34 years old	49	250
	35–54 years old	143	
	55–69 years old	51	
	70 years or older	7	
V.2—Gender	Male	86	250
	Female	164	
V.3—Academic level	None	0	250
	Basic (1st cycle)	5	
	Basic (2nd and 3rd cycles)	14	
	Secondary/postsecondary	43	
	Superior (degree or more)	188	
V.4—Area of residence	*Big* Lisbon	94	250
	*Big* Oporto	111	
	Region of Cávado	45	
V.5—Do you have children under 18 living with you?	Yes	117	250
	No	133	
V.6—What is the best place to purchase certified organic products?	Fairs/producer markets	105	250
	Organic supermarkets	61	
	Generalist super and hypermarkets	52	
	Home delivery baskets	16	
	Traditional shops	16	

**Table 3:** Raw best–worst scores, average best–worst scores, and standardized aggregated importance weights.

Attribute	*N*	Times Selected Best	Times Selected Worst	(*B*-*W*)/*n*	Sqrt (*B-W*)	Standardized Ratio Scale	Standardized Importance Weights (%)	Rescaled Scores Average	95% Lower	95% Upper
Health benefits	7	609.0	15.0	2.376	6.37	100.0	40.1	25.1	24.5	25.6
Absence of GMOs	10	356.0	59.0	1.188	2.46	38.6	15.5	18.6	17.6	19.6
Environmental impact	8	229.0	45.0	0.736	2.26	35.4	14.2	14.2	13.3	15.1
Certification warranty (EU logo)	3	209.0	157.0	0.208	1.15	18.1	7.3	11.2	10.2	12.3
Nutritional value	9	184.0	174.0	0.040	1.03	16.1	6.5	9.8	8.8	10.8
Origin	4	138.0	249.0	−0.444	0.74	11.7	4.7	6.9	6.1	7.8
Expectation of better taste	5	127.0	227.0	−0.400	0.75	11.7	4.7	6.3	5.4	7.0
Price	1	65.0	275.0	−0.840	0.49	7.6	3.0	3.9	3.2	4.6
Availability	6	49.0	361.0	−1.248	0.37	5.8	2.3	2.4	1.9	2.9
More natural appearance	2	34.0	438.0	−1.616	0.28	4.4	1.7	1.6	1.2	1.8

**Table 4:** Sample structure.

Classification Variable	Modality	*N*	
V.1—Age	≤54 years old	192	250
	≥55 years old	58	
V.2—Gender	Male	86	250
	Female	164	
V.3—Academic level	Not superior	62	250
	Superior (degree or more)	188	
V.4—Area of residence	*Big* Lisbon	94	250
	*Big* Oporto and Cávado	156	
V.5—Do you have children under 18 living with you?	Yes	117	250
	No	133	
V.6—What is the best place to purchase certified organic products?	Fairs/producer markets, general supermarket and hypermarkets	157	250
	organic supermarkets, home delivery baskets, traditional stores	93	

**Table 5:** Chi-square test for independence (*p*-values).

	Classification Variable					
Attribute	Age	Gender	Academic Level	Area of Residence	Children under 18 at Home	Place to Purchase Organic Food
	*p*					
Price	0.150	0.508	0.618	0.978	0.694	0.584
More natural appearance	0.472	0.766	0.253	0.941	0.124	0.053 ^b^
Certification warranty (EU logo)	0.748	0.543	0.272	0.492	0.011 ^a^	0.210
Origin	0.502	0.679	0.530	0.947	0.399	0.563
Expectation of better taste	0.682	0.145	0.544	0.929	0.344	0.834
Availability	0.654	1.000	0.378	0.162	0.052 ^b^	0.919
Health benefits	0.528	0.360	0.571	0.854	0.710	0.857
Environmental impact	0.465	0.831	0.731	0.926	0.998	0.721
Nutritional value	0.791	0.860	0.898	0.529	0.964	0.800
Absence of GMOs	0.288	0.377	0.401	0.547	0.951	0.109

## Data Availability

Not applicable.
